# Study on the Mechanism of Diosgenin Targeting STAT3 to Inhibit Colon Cancer Proliferation and Migration

**DOI:** 10.1155/2022/7494887

**Published:** 2022-06-04

**Authors:** Zonglang Lai, Huaibi Wang, Xiaohui Tang, Liufan Zhang, Tiantian Wang, Jun Cheng

**Affiliations:** Department of Oncology, Chongqing Traditional Chinese Medicine Hospital, Chongqing 400021, China

## Abstract

To elucidate regulatory effects and molecular mechanisms of diosgenin on colon cancer, this study administered diosgenin at concentrations of 10 (low), 50 (medium), and 100 *μ*mol/L (high concentration group) at the cell level, respectively. EdU, colony formation, and Transwell assays were implemented to determine SW480 cellular proliferation and migration. Assays of flow cytometry and TUNEL were employed to estimate cell apoptosis. Additionally, nude mouse tumorigenesis assay was used to further verify the regulatory function of diosgenin on colon cancer. The target protein of diosgenin was predicted via molecular docking. The results showed that all three concentrations of diosgenin could reduce colon cancer cellular proliferation and migration, and after diosgenin treatment, colon cancer cellular apoptosis was markedly increased, and the 100 *μ*mol/L diosgenin group produced the most satisfactory inhibition on colon cancer cell proliferation. Ki67 expression was markedly reduced whereas those of Bax and caspase3 were greatly increased after diosgenin treatment. The nude mouse tumorigenesis assay indicated that the parameters of tumorous volume and mass of diosgenin treatment group were greatly decreased as compared to control, and as the concentration of diosgenin increased, the inhibitory effect was more significant. Molecular docking indicated that STAT3 served as a target protein of diosgenin. Moreover, after diosgenin treatment on colon cancer cells, the STAT3 expression was markedly reduced. The STAT3 overexpression would counteract the inhibitory effect of 50 *μ*mol/L diosgenin in both suppressing colon cancer cellular proliferation and migration and promoting apoptosis. Taken together, all our outcomes demonstrated the diosgenin effects in not only inhibiting colon cancer cellular proliferation and migration but also promoting cancerous cellular apoptosis. Diosgenin is a regulatory player in targeting and regulating STAT3.

## 1. Introduction

Malignancies have become a vital public health issue gaining extensive worldwide concerns, and it endangers the well-being of humans. Among all of the malignancies, colorectal cancer (CRC) has become a major tumor and currently accounts for about 10% of cancer-related mortality [1, 2]. Generally, CRC ranks as the third most common digestive malignancy in diagnosis rate worldwide. It is also the third most frequently encountered malignant tumor and the fourth cancer-related cause of death. Statistical reports have revealed that more than half of CRC cases in the world occur in developed countries and regions [3, 4]. In China, statistics of the National Cancer Center have indicated that CRC ranks the fourth top major malignant tumors, third in the number of incidences, and the fifth of death causes from major malignant tumors. From the past to the present, the increase in the incidence of CRC can be attributed to the aged population, certain bad eating habits nowadays, and the risk factors such as smoking, lack of physical exercise, and obesity. Due to unhealthy lifestyles, environmental factors, and multiple influences and interactions, a high incidence of malignant tumors of the digestive system including pancreatic cancer, liver cancer, gastric cancer, and CRC are presented at a younger age and poorer prognosis, which brings about a heavy economic burden to the society and individuals, thus, this disease has attracted widespread attention in the academic circle [5, 6]. With the increasing incidence of malignant tumors, especially in the digestive tract, numerous experts and scholars have focused on the use of traditional Chinese medicine (TCM) to treat malignant tumors of the digestive tract [7]. Significant results have been achieved in both theoretical innovation and clinical application, and to further explore it in the treatment of malignant tumors of the digestive tract with TCM, great priority has been given in the clinical prevention and treatment of precancerous lesions, alleviating side effects caused by radiotherapy and chemotherapy, ameliorating patients' life quality, and prolonging survival period [8, 9].

Diosgenin is known as saponin and belongs to steroidal saponin presenting as white crystals. Diosgenin was first isolated and prepared by Japanese scholars. By 1943, Marker and other scholars have developed a method of low cost, high efficiency, and easy chemical process to degrade steroidal sapogenins [10, 11]. They have pioneered the synthesis of steroid hormone drugs from plant raw materials and laid an important foundation for the further in-depth study of steroidal sapogenins in dioscorea plants. Studies have shown that diosgenin as a steroid hormone has strong pharmacological works including antitumor, antiallergic, antibacterial, viral infection, and antishock. It can treat tumors, rheumatic diseases, heart, and brain, which is an important medication for vascular diseases, acute and chronic lymphocytic leukemia, skin allergies, and rescue shock patients [12–14]. In several cancer cell lines, diosgenin exerts its antitumor effect in multiple ways. Diosgenin and tumor necrosis factor-related apoptosis-inducing ligand (TRAIL) through the mitogen-activated protein kinase (MAPK) signaling transduction pathway or mitochondrial pathway and other mechanisms synergistically promote the apoptosis of nonsmall cell lung cancer A549 cells [15–17]. It appears that the combination of the two drugs promotes apoptosis. In the combination group, the activations of caspase-3, -8, and -9 and the apoptotic proteases Bid and Bax and the activity of polyadenosine diphosphate ribose polymerase (PARP) are markedly increased, whereas the tumor suppressor gene Bcl-2 is decreased, and p38 MAPK, c-Jun N-terminal kinase (JNK), and extracellular signal-regulated kinase (ERK) are obviously activated [10, 18]. Diosgenin may induce cycle arrest of human hepatocelluar carcinoma (HCC) SMMC-7721 cell line by inhibiting the expressions of p21 and p27 upregulated by phosphatidylinositol-4,5-bisphosphate 3-kinase/protein kinase B (PI3K/Akt) and inhibiting the proliferation of human HCC SMMC-7721 cells [19].

The antitumor mechanism of diosgenin has not been totally understood. The present study is aimed at investigating how diosgenin worked on human colon cancer cells and at explicating the effect and the possible mechanism in pharmacology so as to provide new ideas for colon cancer treatment. Signal transducer and activator of transcription 3 (STAT3) is a signaling molecule that promotes the development of colon cancer and is high expressed in colon cancer. It is predicted that diosgenin is likely to be involved in colon cancer proliferation and migration by targeting STAT3.

## 2. Materials and Methods

### 2.1. Cell Culture and Patient Grouping

The cell line we exploited in the study was human colon cancer SW480, which was fetched from Cell Bank of Chinese Academy of Sciences. RPMI1640 medium was added with 10% standard fetal bovine serum and formulated with 100 *μ*g/mL streptomycin and 100 U/mL penicillin medium. Those cells were cultivated in an incubator with the context of 37°C with 5% CO_2_.

Diosgenin was purchased from Xi'an Gaoyuan Biotechnology Co., Ltd., with purity ≥ 98% and CAS no. 512-04-9. The mediums supplemented with different concentrations of diosgenin (10, 50, and 100 *μ*mol/L) were utilized in the cultivation of different groups of cells. In terms of diosgenin concentrations, the cells were grouped, and there were our experimental groups; the group with 40 *μ*mol/L cisplatin was deemed as the positive control, and the one with equal quantity of PBS was the negative control [20].

### 2.2. Cell Transfection Assay

Signal transducer and activator of transcription 3 (STAT3) lentivirus was constructed and packaged by Chongqing Biomedicine Biotechnology Co., Ltd. The cells were transfected at the ratio of liposome volume: DNA mass at 1 : 1. Tubes with transfection agent were taken and added with appropriate volume of serum-free medium, which were placed at room temperature for 10 min. Discarding previous medium, the cells in culture plate were rinsed with PBS or serum-free medium; subsequently, each well with cells in culture plate was added with mixed solution, then, the plate was placed back to incubator for 1 h for cultivation. The mixed solution was removed as soon as the culture was completed. Complete medium was supplemented and continued the culture for 24 h.

### 2.3. Ethynyl-Deoxyuridine (EdU) Staining

EdU kit (SolarBio, China) was utilized for diosgenin's effects on SW480 cellular proliferation: 200 *μ*L of the 3rd generation SW480 cells was grown in 96-well plates in a density of 2 × 10^4^ cells/mL. After cells' adherence, a 72-hour culture was performed with 0.4%-FBS-containing medium for cell cycle synchronization. Subsequently, those cells were separately cultivated with different concentrations of diosgenin (10, 50, and 100 *μ*mol/L) and cisplatin (40 *μ*mol/L); 48 h after that, the medium was removed, cells were incubated with 100 *μ*L of 50 *μ*mol EdU for 2 h. Two hours after that, 4% paraformaldehyde was added into each well for cell fixation. Then, three visual fields were randomly picked on for observation and taken photos with microscopy; each process in this part was conducted repeatedly at least 3 times with 6 multiple holes in each group.

### 2.4. Colony Formation Assay

In this section, cells were, respectively, cultivated with different concentrations of diosgenin (10, 50, and 100 *μ*mol/L) and cisplatin (40 *μ*mol/L). 48 h after that, 0.25% of trypsin was added into the wells for cell digestion, then, the cells were harvested. The harvested cells were then suspended and diluted in gradient multiple. 10 mL of medium was preheated at 37°C, then, the cell suspensions of each group were added into dishes with a density of 200 cells per dish. Those dishes were gently rotated for a full cell dispersion. Afterward, those cells were given a two-week cultivation at least until the object clones were visible in the petri dish. When the cell cultivation termination was performed and the supernatant was removed, the cells were rinsed with PBS twice. Finally, 5 mL of 4% paraformaldehyde was added to fix those cells for 15 min. Following removal of fixation solution, an appropriate GIMSA dyeing solution was added for staining 30 min and slowly washed away the staining solution with running water. The petri dish was inverted and superimposed with a transparent mesh film, and the clones were counted directly with naked eyes.

### 2.5. Transwell Assay

When treated with different concentrations of diosgenin (10, 50, and 100 *μ*mol/L) and cisplatin (40 *μ*mol/L) for 48 h, those cells were given with 0.25% trypsin for digestion and harvested. As 800 *μ*L of 10% FBS medium was added into each well of 24-well plate, the plate was placed into a Transwell chamber (BD Company, USA). By adding 200 *μ*L of cell suspension of each group to the upper Transwell chamber for incubation 48 h at 37°C with 5% CO_2_, the Transwell was then taken out and cleaned once using PBS carefully. The cells were fixed for 1 h with 70% iced ethanol solution, stained with 0.5% crystal violet dye, let standby for 20 min at room temperature, and rinsed with PBS. On one side of the upper chamber, there were some unmigrated cells that were wiped off using a clean cotton ball, visualized and photographed using a microscope, and calculated the number.

### 2.6. Flow Cytometry on Cellular Apoptosis

The cells that were separately cultivated with different concentrations of diosgenin (10, 50, and 100 *μ*mol/L) and cisplatin (40 *μ*mol/L) for 48 h were digested and harvested, suspended in a density of 1 × 10^6^/mL in a 10 mL centrifuge tube. Followed by centrifugation at 1,000 g for 5 min, the culture medium was removed. After a cycle of washing with incubation buffer, the cells were centrifuged at 1,000 g for 5 min, resuspended in 100 *μ*L labeled solution, incubated again for 10 min at room temperature and kept away from light, and centrifuged at 1,000 g for 5 min. After cell precipitation and culture, buffer solution was employed for another cycle of washing. Fluorescence (SA-FLOUS) solution was added for incubation at 4°C for 20 min away from light, vibrating occasionally. The excitation light wavelength was 488 nm by flow cytometry. FITC fluorescence was estimated using a filter at 515 nm wavelength, and PI was also assessed at 560 nm wavelength.

### 2.7. Terminal Deoxynucleotidyl Transferase dUTP Nick End Labeling (TUNEL) Staining

Following three cycles of washing with PBS solution, the samples were added 100 *μ*L protease K with 20 min transparent processing in a wet box, washed again with PBS for culture in 3% H_2_O_2_ solution, and sealed for 10 min. The cells were then added with 100 *μ*L positive incubation solution and incubated in the box at 37°C for 30 min, supplemented with 50 *μ*L labeled reaction solution by dropwise, and culture under the same condition for 90 min. After that, 50 *μ*L PI dye solution was added for reaction at room temperature for 5 min and dried in an oven at 60°C. Section sealing was performed using DAPI antifluorescence quenching agent, and images were captured with a microscope.

### 2.8. Molecular Docking

First, we downloaded the 3D structure of diosgenin from Pubchem (https://pubchem.ncbi.nlm.nih.gov/), conversed from sdf format to mol2 format using OpenBabel 3.1.1, imported into the AutoDock Tools 1.5.7 software, and saved it as pdbqt format. The molecular structure of STAT3 was obtained from RCSB PDB database (https://www.rcsb.org/), the original water molecules in the structure were deleted using PYMOL 2.5.2 software, and additional ligands originally contained in the structure were deleted by combining the protein structure name. A plugin GetBox was used to calculate the spatial coordinates of the docking box. The addition of hydrogen and charges of the protein structure were performed using AutoDock Tools 1.5.7, and output as pdbqt format. The molecular docking was ultimately run by the AutoDock Vina 1.1.2 program. A semiflexible docking method was used for screening the top 10 conformation models with the highest scores in terms of the binding affinity calculated by scoring function, and the conformation with the lowest binding energy was selected for docking.

### 2.9. Western Blot Detection

After lysed with RIPA lysate on ice for 30 min, the cells were carefully scraped and centrifuged at 12, 000 × g for 10 min. The supernatant was obtained and kept at −80°C for later use. Determination on the total protein concentration of the samples was accomplished with BCA method, then, the samples were added with SDS-PAGE loading buffer and bathed in boiling water for 10 min. Proteins were separated in a manner of 10% SDS-PAGE and transferred onto polyvinylidene fluoride (PVDF) membranes. After transference, PVDF membrane blocking was conducted using TBST buffer of 5% skimmed milk at room temperature for 1 h. Next was the addition of primary antibodies for incubation overnight at 4°C. Following three cycles of TBST washing, the PVDF membrane was incubated by supplemented with horseradish peroxidase-labeled secondary antibodies at room temperature for 1 h. After completion of secondary antibody incubation, the membrane was washed again using TBST buffer for 3 times and visualized the chemiluminescence results using a BioRad Gel Imaging System. The Image J software was employed to analyze the gray values of each target band. Using GAPDH as internal reference, gray ratio of target protein to internal reference was calculated, and the protein expression of each group was analyzed.

### 2.10. Colon-Cancer-Tumor-Bearing Nude Mouse Model Establishment and the Grouping

All usages of the experimental animals in this study were in compliance with the demands of the Experimental Animal Ethics Committee of Chongqing Traditional Chinese Medicine Hospital. SPF grade BALB/C nude mice aged 6 weeks, weighing 17.6 ± 2.3 g prior to the experiment. All animals were bought from Chongqing Ensiweier Biological Technology Co., Ltd and kept in a sterile environment. The temperature was at 26-28°C, the humidity was 50-60%, and the light/dark cycle was 12 h. All mice were given unhindered access to food and water. 7 days after the adaptive feeding, the experiment was initiated. First, colon cancer SW480 cell suspension of the density of 2 × 10^7^ cells/mL was prepared, which was subcutaneously injected into the same part in the neck of nude mice, and there was a colon-cancer-tumor-bearing nude mouse model established. On the second day after that, those mice were randomly grouped, and there was a model control group, a positive control group, low-, medium-, and high-dose diosgenin groups, with 10 mice in each treatment. Thereinto, mice of low, medium, and high diosgenin groups were, respectively, given 50, 100, and 200 mg/kg diosgenin in a manner of gavage. Mice of model control group were given with the injection of the same volume of 0.9% sodium chloride. Mice of the positive control group were given an intraperitoneal injection of 3 mg/kg cisplatin; in a frequency of once per day, mice in this group were given with a 4 consecutive-week gavage.

### 2.11. Immunofluorescence

SW480 cells were grown in 24-well plates, replacing the culture medium with 0, 10, 50, and 100 *μ*mol/L diosgenin or 40 *μ*mol/L cisplatin 48 h later, when cell growth at about 60%. The culture medium was subsequently removed. The cells were fixed with precooled methanol at -20°C for 30 min, rinsed using PBS buffer for 3 times, and followed by blocking of PBS buffer containing 5% BSA albumin at room temperature for 2 h. After sealing, PBS buffer was employed again for cell wash 3 times, supplemented with primary antibody, and incubated overnight at 4°C. After the incubation, PBS was used for three cycles of washing. The cells were then supplied with fluorescein isothiocyanate (FITC) labeled secondary antibodies, incubated 40 min at room temperature with three cycles of PBS washing, and visualized under a fluorescence microscope.

### 2.12. Statistical Analysis

In this section, GraphPad Prism 8.0 software was exploited for data statistics and plotting analysis. All the outcomes were presented in the form of mean ± standard deviation (mean ± SD). With the help of one-way ANOVA, the outcomes of the mean analysis on multiple groups were obtained. The one with its *p* value < 0.05 was regarded as statistically significant.

## 3. Results

### 3.1. Diosgenin Inhibits the Proliferation and Migration of Colon Cancer Cells Cultured In Vitro and Promotes Apoptosis of Colon Cancer Cells

**Figure 1:**
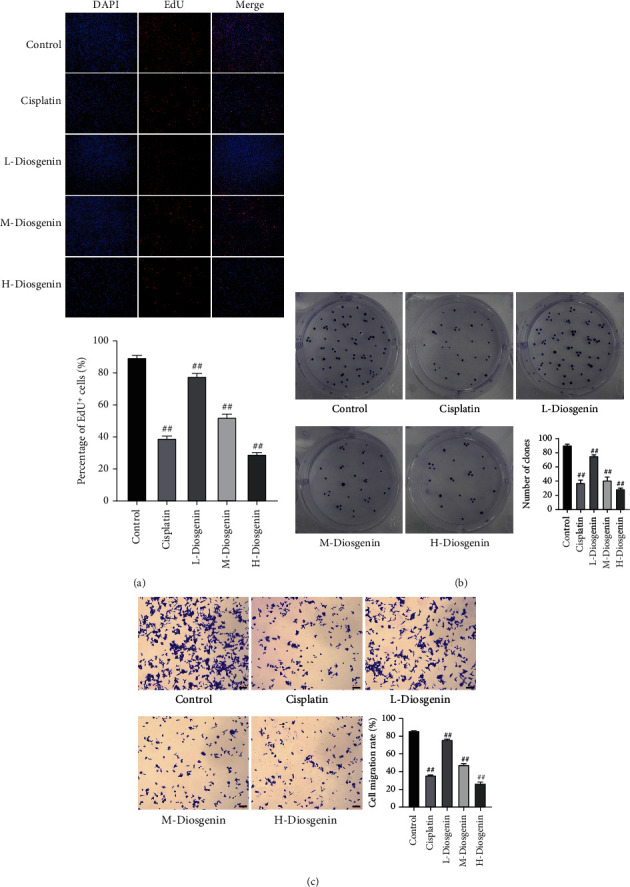
Diosgenin suppresses colon cancer cells' proliferation and migration cultured in vitro. (a) EdU staining to detect cell proliferation. (b) Cell colony formation assay to detect cell proliferation ability. (c) Transwell detection of migration ability of cells. ^##^*p* < 0.01.

To elucidate diosgenin's work on the proliferation and migration of colon cancer cells, this study added 40 *μ*mol/L cisplatin (positive control group), 10 (low), 50 (medium), and 100 *μ*mol/L diosgenin (high concentration group) to SW480 cells cultured in vitro. EdU staining and cell colony formation tests were performed to detect cell proliferation ability. The three concentrations of diosgenin in the experiments could reduce the proliferation rate of colon cancer cells, and the 100 *μ*mol/L diosgenin group generated the most satisfactory inhibition on colon cancer cellular proliferation (Figures 1(a) and 1(b)). The Transwell assay detected cell migration, indicating that the cell migration rate decreased after diosgenin treatment. The 100 *μ*mol/L diosgenin group had the best inhibitory effect on colon cancer cell migration, which was similar to the positive drug cisplatin (Figure 1(c)).

**Figure 2:**
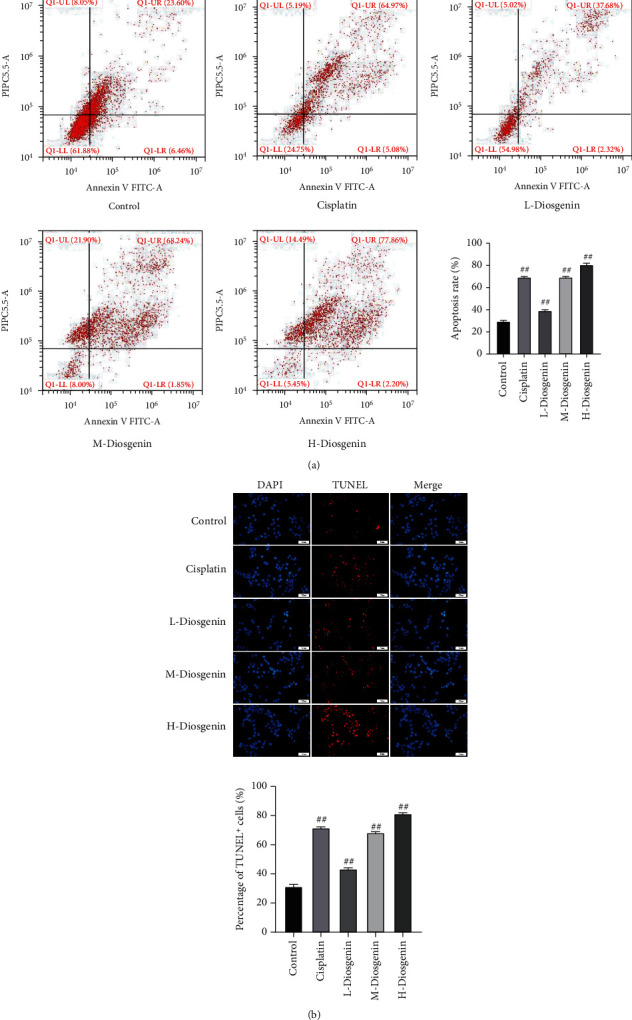
Diosgenin promotes apoptosis of colon cancer cells. (a) Flow cytometry detection of cell apoptosis. (b) Detection of cell apoptosis by fluorescence TUNEL. ^##^*p* < 0.01.

**Figure 3:**
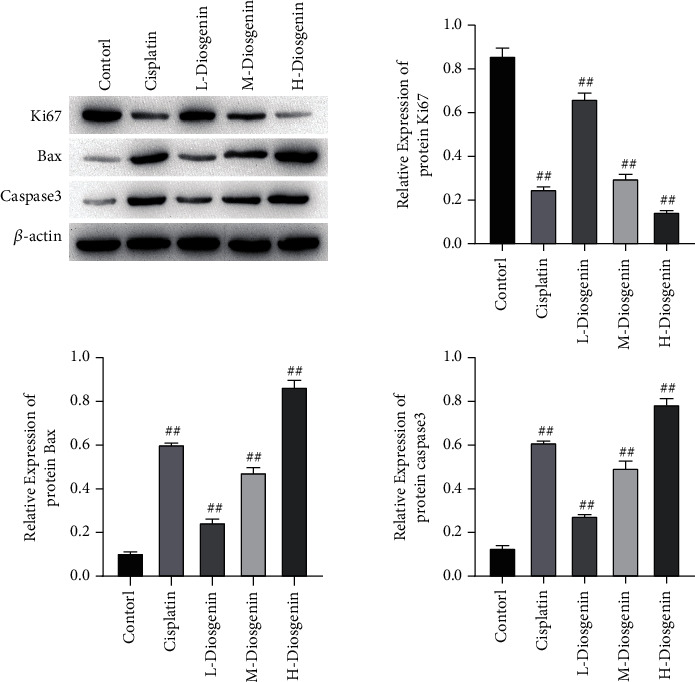
Marker genes associated with proliferation and apoptosis were determined through Western blot. Protein expressions of Ki67, Bax, and Caspase3 were estimated, and gray value analysis was conducted. ^##^*p* < 0.01.

Flow cytometry and fluorescent TUNEL methods were for detecting cell apoptosis. The results showed that all three concentrations of diosgenin could incur apoptosis of colon cancer cells. After 100 *μ*mol/L diosgenin treatment, apoptosis of colon cancer cells reached the highest (Figures 2(a) and 2(b)). Western blot was used to detect marker genes related to proliferation and apoptosis, and it was found that after diosgenin treatment, the expression of Ki67 decreased significantly, and as the concentration increased, the expression gradually attenuated; expressions of Bax and caspase3 increased significantly and upregulated in a diosgenin concentration-dependent manner (Figure 3).

**Figure 4:**
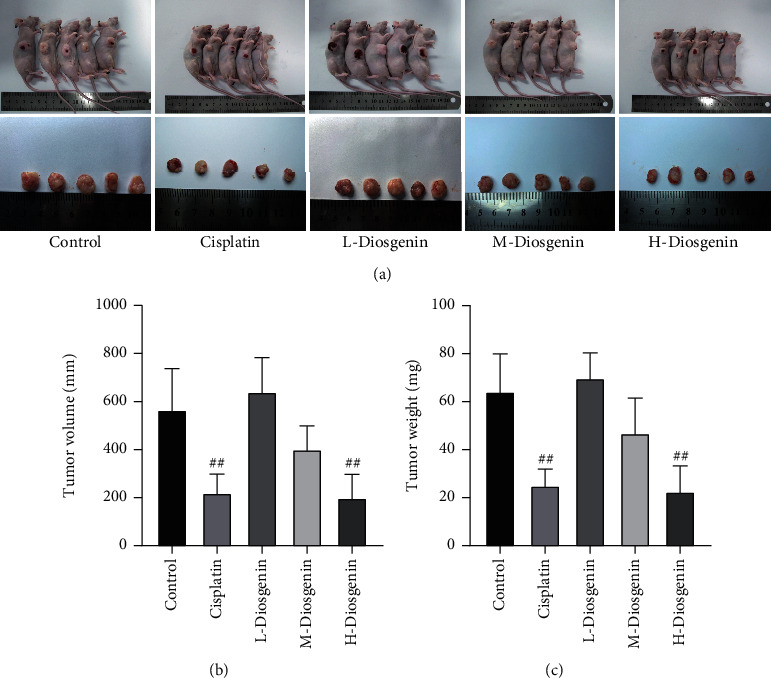
Tumorigenesis in nude mice. Nude mice were inoculated with colon cancer cells and treated with diosgenin in different concentrations for 4 weeks. (a) In vivo and in vitro images of tumors. (b) Tumor volume in nude mice was detected after different treatment. (c) Tumor weight measurement. ^##^*p* < 0.01.

To explicate the inhibitory effect of diosgenin on colon cancer, cells were inoculated into nude mice and treated with diosgenin at different concentrations. After 4 weeks, the volume and quality of the tumor were measured. The conclusions obtained were consistent with the conclusions at the cellular level. The tumor volume and mass of the diosgenin treatment group were obviously decreased as compared with control, and as the diosgenin concentration increased, the inhibitory effect became more significant (Figure 4). The above results indicated that diosgenin could significantly inhibit the development of colon cancer.

### 3.2. STAT3 Is the Target Protein of Diosgenin

**Figure 5:**
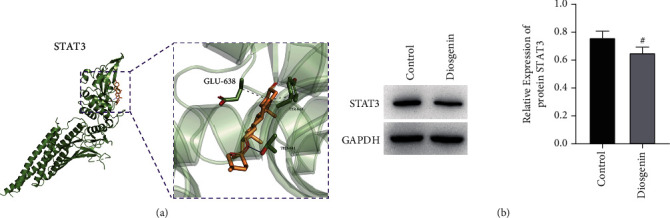
STAT3 is the target protein of diosgenin. (a) Molecular docking simulates diosgenin forms hydrogen bonds with GLU-638, TYR-640, and THR-641 of STAT3. (b) Western blot detection of diosgenin treatment on colon cancer cells indicates a decreased expression of STAT3. ^#^*p* < 0.05.

We predicted that STAT3 might be the target protein of diosgenin through molecular docking. Diosgenin and GLU-638, TYR-640, and THR-641 of STAT3 were predicted to form hydrogen bonds (Figure 5(a)). STAT3 is known to be high-expressed in colon cancer and is a signaling molecule that promotes the development of colon cancer. Western blot detection showed that after adding diosgenin to treat colon cancer cells, the expression of STAT3 was largely reduced (Figure 5(b)). In general, STAT3 acted as the target protein of diosgenin, and diosgenin could reduce the expression of STAT3.

### 3.3. Cotransfection Experiments Verify That Diosgenin Affects the Development of Colon Cancer by Regulating STAT3

**Figure 6:**
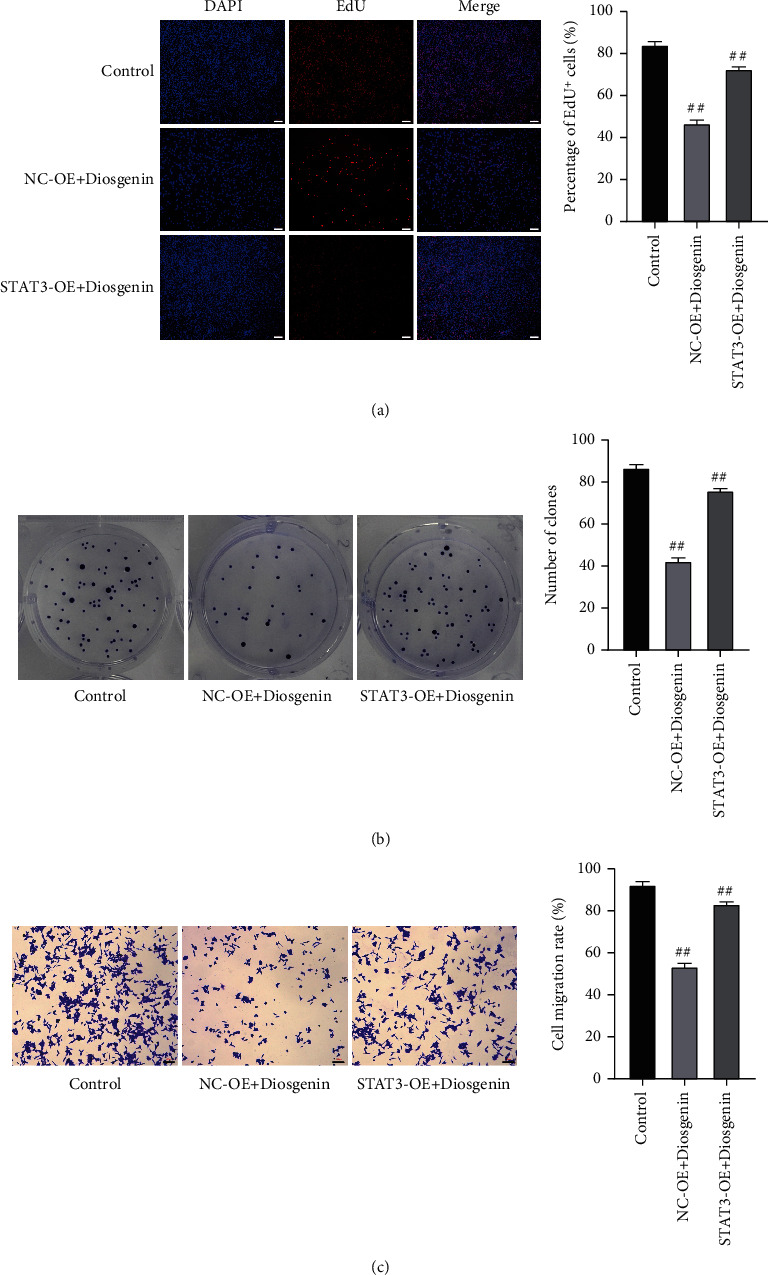
Condition of cellular proliferation after the co-transfection with 50 *μ*mol/L diosgenin and STAT3 into colon cancer cells. (a) EdU staining to detect cell proliferation. (b) Cell colony formation assay to detect cell proliferation ability. (c) Transwell detection of migration ability of cells. ^##^*p* < 0.01.

**Figure 7:**
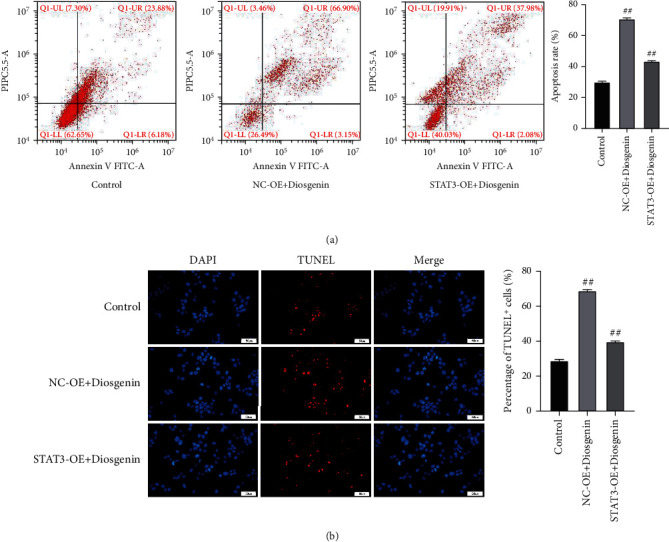
Condition of cellular apoptosis after colon cancer cells subject to the co-transfection with 50 *μ*mol/L diosgenin and STAT3. (a) Flow cytometry detection of cell apoptosis. (b) Detection of cell apoptosis by fluorescence TUNEL. ^##^*p* < 0.01.

**Figure 8:**
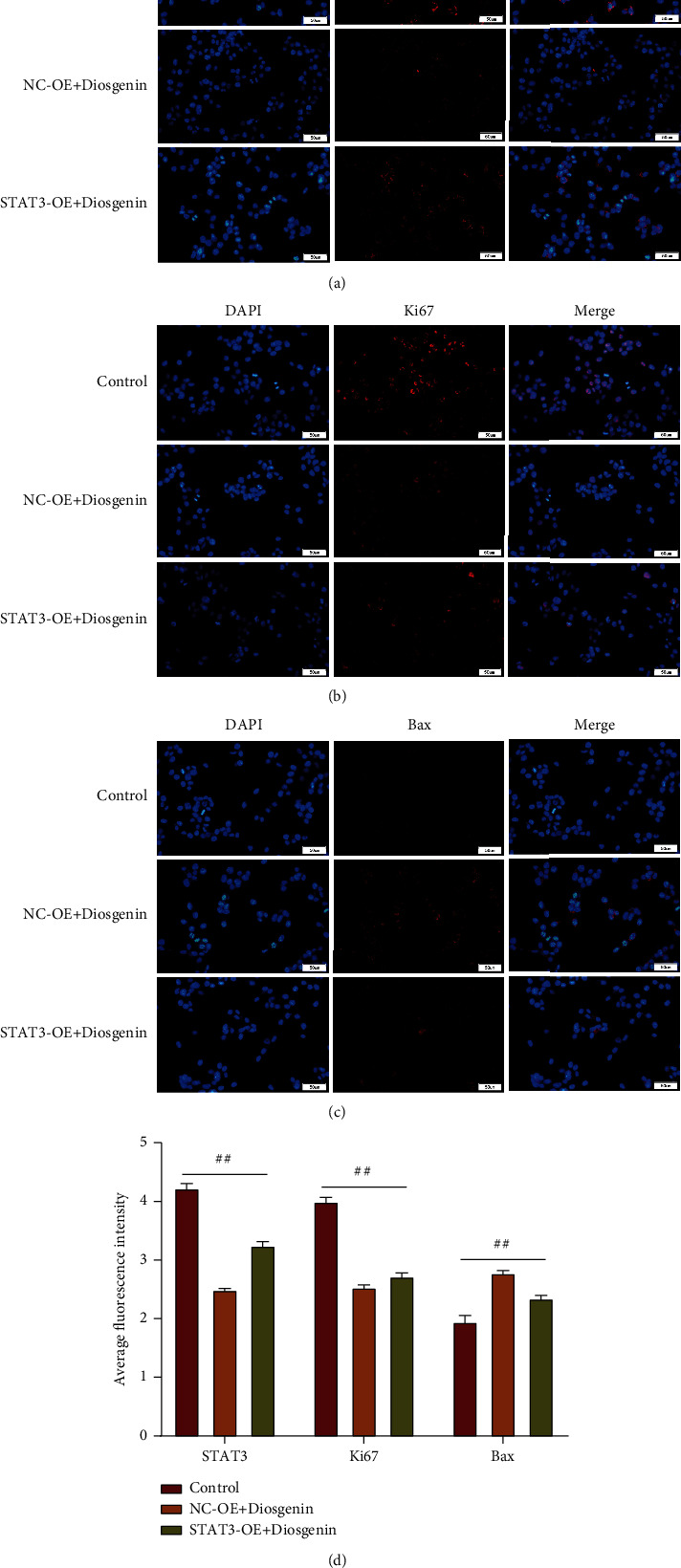
Expression of STAT3, Ki67, and Bax by immunofluorescence after colon cancer cell subject to the cotransfection with 50 *μ*mol/L diosgenin and STAT3. (a) Immunofluorescence assay detection of STAT3. (b) Immunofluorescence assay detection of Ki67. (c) Immunofluorescence assay detection of Bax. (d) Average fluorescence intensity of STAT3, Ki67, and Bax was calculated.

Subsequently, in this study, 50 *μ*mol/L diosgenin and STAT3 were cotransfected into colon cancer cells cultured in vitro to detect the regulatory effect of diosgenin on STAT3 and its influence on colon cancer cells. The results showed that after 50 *μ*mol/L diosgenin and STAT3 overexpressing lentivirus were cotransfected, the EdU positive rate of colon cancer cells was restored, which was notably upregulated compared with the control virus cotransfection group (Figure 6(a)). In addition, the results of cell cloning and cell migration were also consistent (Figures 6(b) and 6(c)), indicating that further overexpression of STAT3 would offset the inhibitory effect of 50 *μ*mol/L diosgenin on colon cancer cells. Flow cytometry and TUNEL findings indicated that colon cancer cell apoptosis was inhibited after 50 *μ*mol/L diosgenin was cotransfected with overexpressing STAT3 and overexpressing lentivirus. After overexpression of STAT3, it would offset the apoptosis of colon cancer cells promoted by 50 *μ*mol/L diosgenin (Figures 7(a) and 7(b)). Detecting the expression levels of STAT3, Ki67, and Bax by cell immunofluorescence revealed that diosgenin could significantly reduce the expressions of STAT3 and Ki67 and increased the expression level of Bax. The overexpression of STAT3 cotransfection group reversed the regulation effect of diosgenin on the abovementioned proteins to a certain extent (Figures 8(a)–8(c)).

## 4. Discussion

Colon cancer, the third major malignancy worldwide, holds a high incidence and fatality rate. Its occurrence correlates with cell proliferation, apoptosis, metastasis, autoimmune system, and tumor microenvironment [21, 22]. In this study, in vitro cultured colon cancer cells and nude mouse tumorigenesis models; the regulation of diosgenin on colon cancer cellular proliferation, migration, and apoptosis was tested; and the molecular regulation mechanism of diosgenin was studied.

Treatment of TCM strategy on the malignancy of digestive tract proves to run through the whole disease treatment process. The therapy can either contain single TCM drugs or TCM drugs combined with Western medicine. The TCM option alone as a palliative treatment is suitable for patients who cannot tolerate surgical treatment or radiotherapy and chemotherapy. The main characteristic of TCM treatment is syndrome differentiation and treatment, while the main characteristic of Western medicine treatment is evidence-based differentiations [10, 23]. The combination of both not only considers the patient's overall condition but also targets the specific lesions of patients' disease conditions. Integrated TCM and Western medicine treatment is mainly applied for patients who underwent surgeries or chemoradiation therapy, allowing to attenuate the side effects of radiotherapy or chemotherapy or surgery, improve the efficiency of radiotherapy and chemotherapy, and ameliorate patients' quality of life. For patients who have undergone radical surgeries or completed radiotherapy and chemotherapy, TCM treatment can also improve the body's immunity and inhibit tumor recurrence and metastasis. Recent studies have shown that the anticancer and cancer inhibitory theory of TCM mainly include antiangiogenesis, tumor cell proapoptosis, cytotoxicity, extracellular matrix antidegradation, improvement of body immunity, participation in regulation of cell signal transduction, and the attenuation and synergistic effect of radiotherapy and chemotherapy [24, 25]. Chongqing Traditional Chinese Medicine Hospital is a special hospital of TCM. This unit boasts a long history in the clinical application and basic research of Chinese herbs and traditional Chinese medicine, and it has developed a variety of in-hospital preparations for the treatment of tumors.

Targeting the STAT3 signaling pathway has been a promising therapeutic strategy for numerous cancers, for it is the convergence of numerous oncogenic signaling pathways playing a vital role in regulating the antitumor immune response [26]. The study has reported that the potential role of STAT3 in mediating therapeutic resistance and demonstrated for the first time that STAT3 represents a promising novel molecular target for sensitizing drug-resistant rectal cancer to chemoradiotherapy [27]. Berthenet et al. found that heat shock protein 110 (HSP110) contributes to CRC growth through STAT3 activation [28]. STAT3 inhibition by the novel inhibitor bruceantinol exhibits an effective antitumor effect in vitro and vivo, which proves STAT3 inhibitor bruceantinol can be used for the treatment of human CRC [29]. Moreover, overexpressed and persistently activated STAT3 protein found in CRC cells is observed to contribute to the chemoresistance, initiation, progression, and metastasis of CRC [30].

As a steroidal saponin, diosgenin is characterized by antihyperlipidemia, relaxation of blood vessels, protection of myocardium cells, antiallergy, estrogen-like effects, antidermatological, and antioxidant effects. It also produces significant antitumor and cytotoxic effects on multiple malignant tumor cells. It is manifested in inhibiting the proliferation of malignant tumor cells, inhibiting migration and invasion, inducing apoptosis, and blocking cell cycle. Related studies have shown that on human colon cancer HT-29 cells, diosgenin promotes cell apoptosis in a way of repressing Bcl-xl expression, an antiapoptotic protein, and promoting the phosphorylation and the activation of Caspase-6, an apoptotic protein. Diosgenin is capable of inducing apoptosis of human colon cancer cells HT-29 and HCT116 through the cyclooxygenase-2 and 5-lipoxygenase pathways [31]. It regulates several transcription-related genes, including upregulating the expression of interferon-*γ* receptor, membrane-associated calcium-independent phospholipase A, thereby affecting nucleic acid metabolism and poorly differentiated human stomach [32]. Mucinous adenocarcinoma MGC-803 cells play a role in inducing apoptosis, inhibiting migration and invasion. Diosgenin inhibits signal transduction and STAT3 activation in human HCC cells; inhibits activities of molecules nonreceptor tyrosine kinases, JAK1, and JAK2; and downregulates SH-PTP2 protein expression, thereby inhibiting HCC cell proliferation and promoting its apoptosis [33]. In estrogen-positive (ER+) human breast cancer Bca cells, diosgenin inhibits serine/threonine protein kinase (Akt) activity and also pAkt expression and inhibits the expression of apoptosis-inhibiting proteins Survivin and X-linked inhibitor of apoptosis protein, thereby inhibiting the proliferation of Bca cells [34]. Diosgenin increases the phosphorylation level of JNK to inhibit the proliferation of the breast cancer cells with a high expression of human epidermal growth factor receptor and to promote cell apoptosis, possibly by inhibiting Akt and mTOR phosphorylation [35]. Diosgenin inhibits the proliferation, migration, and invasion of PCa cell PC-3 in by suppressing the expression of VEGF and reducing the activity of matrix metalloproteinases [25, 36]. In human chronic myelogenous leukemia K562 cells and HEL cell apoptosis studies, it has been found that G0/G1 cells are more prone to apoptosis, and diosgenin can increase the expressions of thromboxane synthase (TxS) and cyclooxygenase-2 and induce HEL cell differentiation [37–39]. The antitumor effects of diosgenin are mainly reflected in its traits in both promoting tumorous cellular apoptosis and inhibiting proliferation; this possible mechanism mainly works out along with the regulation of the p38 and JNK signaling pathways.

We found that 10 (low), 50 (medium), and 100 *μ*mol/L (high concentration group) diosgenin could exert an inhibitory effect in both colon cancer cells' proliferation and migration. After saponin treatment, colon cancer cell apoptosis was greatly increased; among them, 100 *μ*mol/L diosgenin group had the best inhibitory effect on colon cancer cell proliferation. Diosgenin and STAT3 formed hydrogen bonds. After adding diosgenin to treat colon cancer cells, the expression of STAT3 was significantly reduced. Cotransfection experiments showed that STAT3 counteracted not only diosgenin's effect in inhibiting colon cancer cells' proliferation and migration but also that in promoting apoptosis. In this study, diosgenin's inhibitory effect on colon cancer was consistent with the results in previous studies upon other tumors, indicating that the application of diosgenin in tumor treatment might have a potential prospect.

In future research, we will thoroughly explore the molecular regulation mechanism of diosgenin. After knocking out STAT3 at the animal level, we will add diosgenin treatment to observe the development of colon cancer in animal models. In addition, we also consider the changes in the metabolism of taking diosgenin in clinical patients and analyze the regulation mechanism of diosgenin more comprehensively.

## 5. Conclusions

Diosgenin suppressed both proliferation and migration of colon cancer cells; besides that, it also prompted the apoptosis. The 100 *μ*mol/L diosgenin group had the best effect. Diosgenin and GLU-638, TYR-640, and THR-641 of STAT3 were predicted to have hydrogen bonds. Overexpression of STAT3 withdrew not only the diosgenin's effect in suppressing both colon cancer cells' proliferation and migration but also in apoptosis promotion.

## Data Availability

The data used to support this research are included within this article.
